# Diagnosis of true aneurysms of subclavian and axillary arteries and our principles of surgical approach

**DOI:** 10.1186/1749-8090-8-S1-P46

**Published:** 2013-09-11

**Authors:** O Gokalp, I Yurekli, L Yilik, U Yetkin, T Gunes, M Akyuz, B Ozcem, O Tetik, G Ilhan, A Gurbuz

**Affiliations:** 1 Izmir Katip Celebi University Ataturk Training and Research Hospital, Department of Cardiovascular Surgery, Turkey

## Background

Although pseudoaneurysms of the subclavian and axillary arteries are relatively common due to trauma and diagnostic/therapeutic interventions, true aneurysms of these arteries are rarely seen.

## Methods

Eight patients that were operated on between February 1998 and December 2007 due to true aneurysms of the subclavian and axillary arteries were examined retrospectively in terms of clinical and perioperative parameters.

## Results

All the patients underwent selective peripheral arteriography, colored Doppler ultrasound and contrasted thoracoabdominal computed tomography (CT). All the patients were operated on under general anesthesia. Subclavicular approach was preferred except for the case operated on for pseudoaneurysm repair. In this particular case, supraclavicular approach was used for the 1st operation and supraclavicular incision plus a partial median sternotomy for the 2nd. Generally, if the calibration of the saphenous vein graft was appropriate for the arterial segment, this venous graft was used. Otherwise, an artificial graft was interposed. If acute ischemia was coexistent, brachial embolectomy was also performed.

## Conclusion

Isolated aneurysm of subclavian or axillary artery is very rarely seen among all peripheral arterial aneurysms. The true aneurysms of these particular arteries are even more seldom. One should be very careful in the diagnosis and treatment processes.

